# Optimization of lipase production on agro-industrial residue medium by *Pseudomonas fluorescens* (NRLL B-2641) using response surface methodology

**DOI:** 10.1080/13102818.2014.991635

**Published:** 2014-12-16

**Authors:** Mehtap Tanyol, Gülşad Uslu, Vahap Yönten

**Affiliations:** ^a^ Department of Environmental Engineering, Tunceli University, Tunceli, Turkey; ^b^ Department of Environmental Engineering, Firat University, Elazig, Turkey; ^c^ The Technology of Chemistry, Vocational School, Tunceli University, Tunceli, Turkey

**Keywords:** *Pseudomonas fluorescens*, optimization, RSM, lipase, sunflower oil cake

## Abstract

The aim of our research was to explore the most cost-efficient and optimal medium composition for the production of lipase from *Pseudomonas fluorescens* (NRLL B-2641) culture grown on sunflower oil cake (SuOC) by applying response surface methodology (RSM). The oil cake was used instead of carbon sources. Peptone, ammonium sulphate and the carbon source (SuOC) were the most important factors as it is obligatory for microbial growth. Subsequently, the optimum values for the carbon source, peptone and ammonium sulphate were found to be 11.10% (w/v), 1.18% (w/v) and 0.83% (w/v), respectively. Experiments carried out under optimum conditions revealed a maximum lipase activity of 10.8 U mL^−1^, which was achieved after 48 h of fermentation. The obtained results were finally verified with batch experiments carried out under the optimum conditions evaluated and it was demonstrated that the SuOC from agro-industrial residue as substrates can be used as an inexpensive base (carbon source) for the production of lipase by *P*. *fluorescens* (NRLL B-2641).

## Introduction

In the present scenario, the most valuable and economically assisted industrial enzymes are hydrolytic enzymes, which include carbohydrates (amylases and celluloses), proteins (proteases) and fat (lipases) hydrolysing enzymes.[1] Lipases of triacylglycerol hydrolases are a class of enzymes, termed as carboxyl esterases, which catalyse the hydrolysis and synthesis to form esters from glycerol and long-chain fatty acids at the lipid–water interface. During hydrolysis, lipases single out the acyl group from glycerides forming lipase–acyl complex, which then transfers its acyl group into the O–H group of water.[2] These reactions are reversible, so the lipases also catalyse the formation of acylglycerols from glycerol and fatty acids.[3]

Lipases have a wide range of substrate specificities, high enantioselectivity and do not require cofactors. Moreover, they act under very mild conditions and are stable at high temperatures and in the presence of organic solvents.[4] Therefore, they serve as versatile tools for industrial biocatalysis, and a large number of current and potential applications have been reported in foods,[5] pharmaceuticals,[6] cosmetics,[7] pulps and paper industries,[1] manufacturers of detergents and pesticides,[8] environmental management,[9] wastewater treatment [10] and biodiesel production.[11]

Although submerged fermentation (SmF), widely used in the enzyme industry, has advantages in process control and good yields of extracellular enzymes, the products in fermented beer are relatively dilute and therefore the downstream process results in high volume of effluents (sewage).[12] In recent years, solid substrate fermentation (SSF) has shown much promise in the development of bioprocesses and products. SSF has been known for centuries and is used successfully in the production of oriental foods. More recently, it has gained importance in the production of microbial enzymes due to several economic advantages over conventional SmF.[13] SSF processes are therefore of special economic interest for countries with an abundance of biomass and agro-industrial residues, as these can be used as cheap raw materials.[13,14]

The residual cakes of oil extraction processes are usually used as animal feed, since they are good sources of protein. Many studies have evaluated the use of these agro-industrial residues as substrates in bioprocesses. The biotechnological application of sunflower, soybean, palm, olive, coconut, mustard, cotton and canola cakes has permitted the production of enzymes, antibodies, biopesticides, vitamins, etc. The cakes are used as substrates in a production medium.[15]

Microorganisms are used for production of lipase.[4] The production of lipases with SSF by *Pseudomonas* species has been studied by various researchers.[16–20] However, almost all literature on SSF refers to bacterial systems; to our knowledge, this is the first report of lipase production by SSF using *Pseudomonas fluorescens*. The present work describes the production of lipase by SSF of sunflower oil cake (SuOC).

Response surface methodology (RSM) is an effective statistical technique commonly used for optimization of multivariable systems. It uses quantitative data in experimental design to determine and simultaneously solve multivariate equations in order to optimize processes or products.[21,22] Usually, this process employs a low-order polynomial equation in a pre-determined region of the independent variables.[23] If there is a curvature in the response, then a polynomial of higher degree, such as a second-order model, must be used to approximate the response, which is later analysed to locate the optimum values of independent variables for the best response value.[21,24] RSM has been successfully carried out for modeling and optimization in anaerobic bioconversion of complex substrates,[25] for developing, improving and optimizing of lactose utilization in whey permeate,[26] for optimization of butylgalactoside synthesis by β-galactosidase from *Aspergillus oryzae*,[27] utilized for maximization of lipid production by *Rhodotorula gracilis*.[28]. Otherwise, xylitol production by *Candida guilliermondii*,[29] medium composition to increased production of C-phycocyanin by *Phormidium ceylanicum*,[30] lipase production by *Candida* sp. [31] and κ-carrageenase production by *Pseudomonas elongate* [32] were effectively carried out by RSM.

The present study aimed to optimize the medium composition to produce lipase from *P. fluorescens* culture grown on SuOC by applying RSM. Central composite design (CCD) was utilized to determine the optimum conditions for maximum lipase activity of the *P. fluorescens* culture.

## Materials and methods

### Microorganism and inoculums

The strain *P. fluorescens* (NRLL B-2641) obtained from the American Type Culture Collection was used in this study. It was maintained on potato dextrose agar (PDA) slant and stored at 4°C. A basal culture was prepared by transferring this stock culture to the nutrient medium containing the following ingredients (in g L^−1^): glucose, 5; yeast extract, 1; peptone, 1; K_2_HPO_4_, 0.5; KH_2_PO_4_, 0.5; (NH_4_)_2_SO_4_, 0.5; MgSO_4_·7H_2_O, 0.005; pH 7.0. The cultivation was performed at 30 °C in an orbital shaker at 150 rpm for 24 h. This culture was used as inoculum in further studies by freshly preparing before each fermentation.

### Substrate

The substrate (as carbon source) used in all experiments was SuOC from the same batch obtained from a local sunflower oil manufacturer (Doga Food Ltd., Manisa, Turkey). The oil cake is the residue of sunflower oil extraction after cold press and solvent extraction. The ground agro-industrial residue was washed twice with distilled water and then dried at 60 °C for 2 days. The particle sizes were chosen between 20 and 100 mesh. The oil cake was stored at 4 °C until the moment of utilization.

### Batch fermentation

The fermentation medium contained 5%–15% (w/v) carbon, 0%–2% (w/v) peptone and 0%–1% (w/v) ammonium sulphate (AS). The solutions were prepared with tap water. The pH of the medium was initially adjusted to 6 ± 0.04 and allowed to follow its natural course throughout the fermentation. For flask cultivations, the fermentations were carried out in 250-mL Erlenmeyer flasks containing 100 mL fermentation medium. After sterilization, each flask was injected with 10% (v/v) inoculum and incubated at 150 rpm in an orbital shaker at 30 °C. Fermentation samples were removed after 48 h. The cell-free supernatant obtained by centrifugation at 5000 rpm for 10 min was assayed for lipase activity.

### Lipase activity determination

Lipase activity was determined titrimetrically using an olive oil emulsion method without the addition of surfactants according to the method of Rosu et al. [33] with some modifications. One millilitre of olive oil was incubated with 3 mL of 100 mM Tris–HCl, pH 8, 0.5 mL 100 mM CaCl_2_ and 5 mL distilled water, and stirred at 37 °C for 10 min. One millilitre of enzyme solution was added to give a final volume of 10.5 mL. After 20 min, the reaction was stopped by adding 20 mL of acetone/ethanol solution (1:1). The amount of free fatty acid was titrated with 0.01 N NaOH solution to pH 10. Blank samples were treated similarly. One unit of extracellular lipase activity (U) was defined as the amount of enzyme necessary to release 1 μmol of fatty acid per minute under the assay conditions.

### Experimental design and statistical analysis

RSM is a collection of mathematical and statistical techniques that are useful for modelling and analysis of problems in which a response of interest is influenced by several variables and the objective is to optimize that response.[22] Once the ranges and interval of the significant factors were decided, RSM was used to determine the optimum magnitude of the factors with respect to lipase activity.

A CCD with three factors (*X*_1_, *X*_2_, *X*_3_) at five levels was conducted in the present work, where *X*_1_ is the carbon source (C), *X*_2_ is peptone (P) and *X*_3_ is AS concentration. The total number of experiments was 20 = 2*k* + 2*^k^* + 6, where *k* is the number of factors. Fourteen experiments were augmented with six replications at the centre points to evaluate the pure error. Table 1 shows the levels of the significant factors tested in CCD, whereas Table 2 shows the experimental design and results of CCD. The first four columns of Table 2 show run number and experimental conditions of the runs. Optimization of the process was evaluated by analysing the *Y*, which was the lipase activity after 48 h in the fermentation medium. In the optimization process, the response can be related to selected factors in quadratic models. A quadratic model is supposed to be as follows [34,35]:
(1) 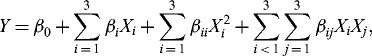
where *Y* is the response, β_0_ is the constant coefficient, *X_i_* (*i* = 1–3) are variables, β*_i_*, β*_ii_* and β*_ij_* (*i* and *j* = 1–3) are the linear, quadratic and second-order interaction coefficients, respectively. Data were processed using the Design-Expert 6.0 program (trial version) and an analysis of variance (ANOVA) test was conducted to obtain the interaction between the process variables and the response. The quality of fit of the polynomial model was expressed by the coefficient of determination *R* ^2^, and its statistical significance was checked by the *F*-test.

**Table 1.** Coded and actual values of independent factors.

		Coding				
Variables	Symbols	−1.682	−1	0	1	+1.682
Carbon source (C)	*X*_1_	5	7.03	10	12.97	15
Peptone (P)	*X*_2_	0.0	0.41	1	1.59	2.0
Ammonium sulphate (AS)	*X*_3_	0.0	0.2	0.5	0.8	1.0

**Table 2.** Parameters, their intervals in the runs conducted in CCD and the corresponding results.

Run	*X*_1_ (%, w/v)	*X*_2_ (%, w/v)	*X*_3_ (%, w/v)	*Y* (U mL^−1^)
1	5.00	1.00	0.50	6.13
2	10.00	0.00	0.50	4.70
3	10.00	1.00	0.50	8.03
4	10.00	1.00	0.50	8.10
5	7.03	0.41	0.80	6.93
6	10.00	1.00	1.00	7.88
7	10.00	1.00	0.50	7.95
8	7.03	1.59	0.80	10.00
9	7.03	1.59	0.20	5.15
10	12.97	0.41	0.20	4.93
11	10.00	1.00	0.00	3.80
12	10.00	1.00	0.50	7.96
13	12.97	1.59	0.20	6.75
14	7.03	0.41	0.20	3.08
15	10.00	1.00	0.50	8.03
16	12.97	0.41	0.80	7.85
17	10.00	2.00	0.50	8.40
18	15.00	1.00	0.50	6.02
19	10.00	1.00	0.50	7.90
20	12.97	1.59	0.80	10.08

### Determination of maximum points

The second-order model obtained from CCD studies (Equation (1)) is adequate for the optimal points. A general mathematical expression (Equation (2)) was used to locate the stationary points.[21,34,35] Writing the second-order model in matrix notation, we have
(2) 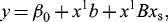
where

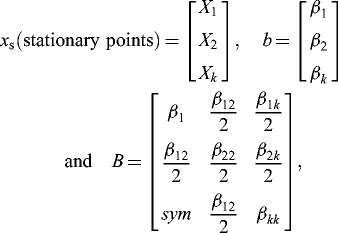
where *b* is a vector of the first-order regression coefficient and *B* is a symmetric matrix whose main diagonal elements are the pure quadratic coefficients (*β_ii_*) and whose off-diagonal elements are one-half of the mixed quadratic coefficients (*β_ij_*, *i* ≠ 1). The stationary points (*x*_s_) are the solution of Equation (3).
(3) 
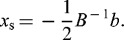



## Result and discussions

The present study particularly focuses on the effects of fermentation medium compositions and conditions on lipase activity. All experiments were carried out in random order as suggested by many design procedures. In the experimental design, the optimum conditions were defined as the operating conditions which maximized the lipase activity of *P. fluorescens* (NRLL B-2641).

It is quite difficult to predict the effects of all parameters in biological processes distinctively, as they may have multiple interactions with one another. Therefore, RSM was applied in order to construct an empirical model for modelling the lipase activity by analysing the effects of the significant factors. The experiments were carried out in random order as suggested in many design programs.[22,31,35]

In the experimental design, optimum conditions were taken to mean the operating conditions required for the maximization of lipase activity of *P. fluorescens* (NRLL B-2641) culture. Table 1 indicates the level of the selected factors designed for CCD, whereas Table 2 shows the experimental conditions for batch shaking-flask runs and the results (responses) in terms of lipase activity. Due to the nature of the RSM, the fermentation medium composition was randomly changed and the corresponding responses were recorded. The observed results are given in Table 2. By applying multiple regression analysis on the experimental data, the following second-order polynomial equation was established to explain lipase activity in terms of medium factors, which constitute the carbon source, peptone and AS (Equation (4)).
(4) 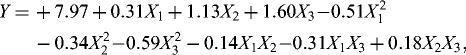
where *Y* is the lipase activity, *X*_1_ is the carbon source (C), *X*_2_ is peptone (P) and *X*_3_ is AS concentration. The ANOVA test was conducted with experimentally observed data to test the significance of the second-order polynomial equation (Equation (4)) and the test results are presented in Table 3. The model *F*-value of 15 implies that the model is significant. The fit of model was checked by the coefficient of determination *R* ^2^, which was calculated to be 0.93, indicating that 93% of the variability in the response could be explained by the model. It indicates a good agreement between the experimental and predicted values and implies that the mathematical model is reliable for predicting lipase activity. The value 0.001 of ‘Prob > *F*’, which is less than 0.05, indicates that the model terms are significant. According to the results of the statistical design and by application of Equations (2) and (3), the optimum values of tested factors were evaluated as follows: 11.1% (w/v) carbon, 1.18% (w/v) peptone and 0.83% (w/v) (NH_4_)_2_SO_4_. Under the optimized conditions, the maximum lipase activity was predicted to be 10.8 U mL^−1^.[36,37]

**Table 3.** ANOVA of second-order polynomial model for the lipase activity obtained through CCD.

Source	Sum of squares	Degree of freedom	Mean square	*F*-value	Prob > *F*	
Model	63.58	9	7.06	14.998	0.0001	Significant
*X*_1_	1.33	1	1.33	2.8278	0.1236	
*X*_2_	17.39	1	17.39	36.929	0.0001	
*X*_3_	34.83	1	34.83	73.959	<0.0001	
*X*_1×2_	0.14	1	0.14	0.315	0.5868	
*X*_1×3_	0.75	1	0.75	1.592	0.2355	
*X*_2×3_	0.24	1	0.24	0.527	0.4843	
*X*_1_^2^	3.73	1	3.73	7.939	0.0182	
*X*_2_^2^	1.68	1	1.68	3.567	0.0882	
*X*_3_^2^	5.05	1	5.05	10.740	0.0083	

Note: *R* ^2^ = 0.93.

**Figure 1.**
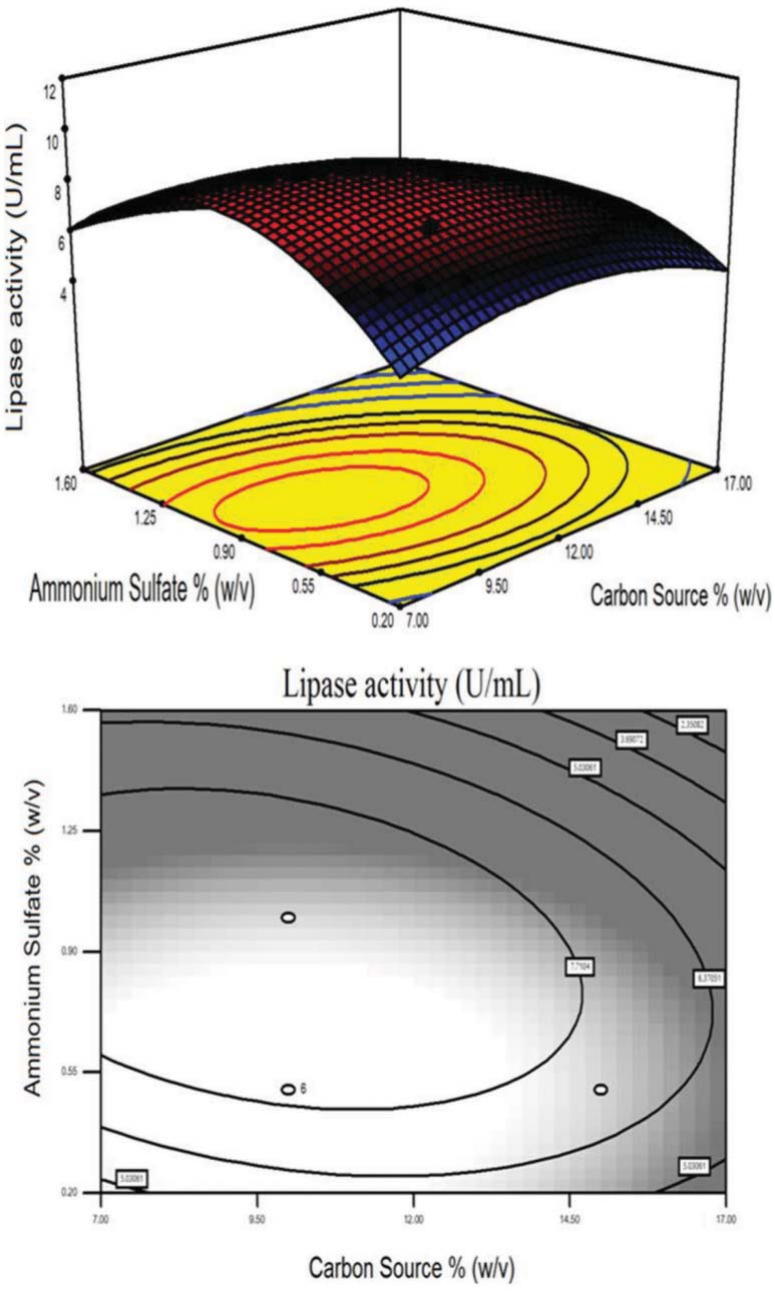
Response surface plot and the corresponding contour plot representing the effects of carbon source and ammonium sulphate concentration on lipase activity at a fixed peptone concentration of 1.0% (w/v).

**Figure 2.**
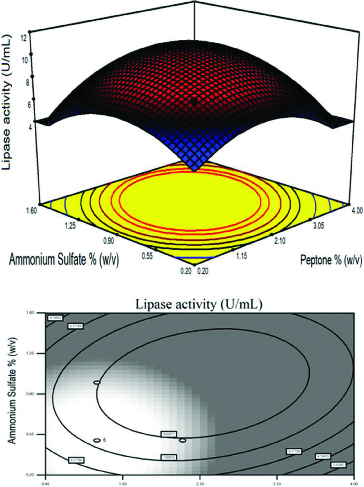
Response surface plot and the corresponding contour plot representing the effects of peptone and ammonium sulphate concentration on lipase activity at a fixed carbon source concentration of 10.0% (w/v).

**Figure 3.**
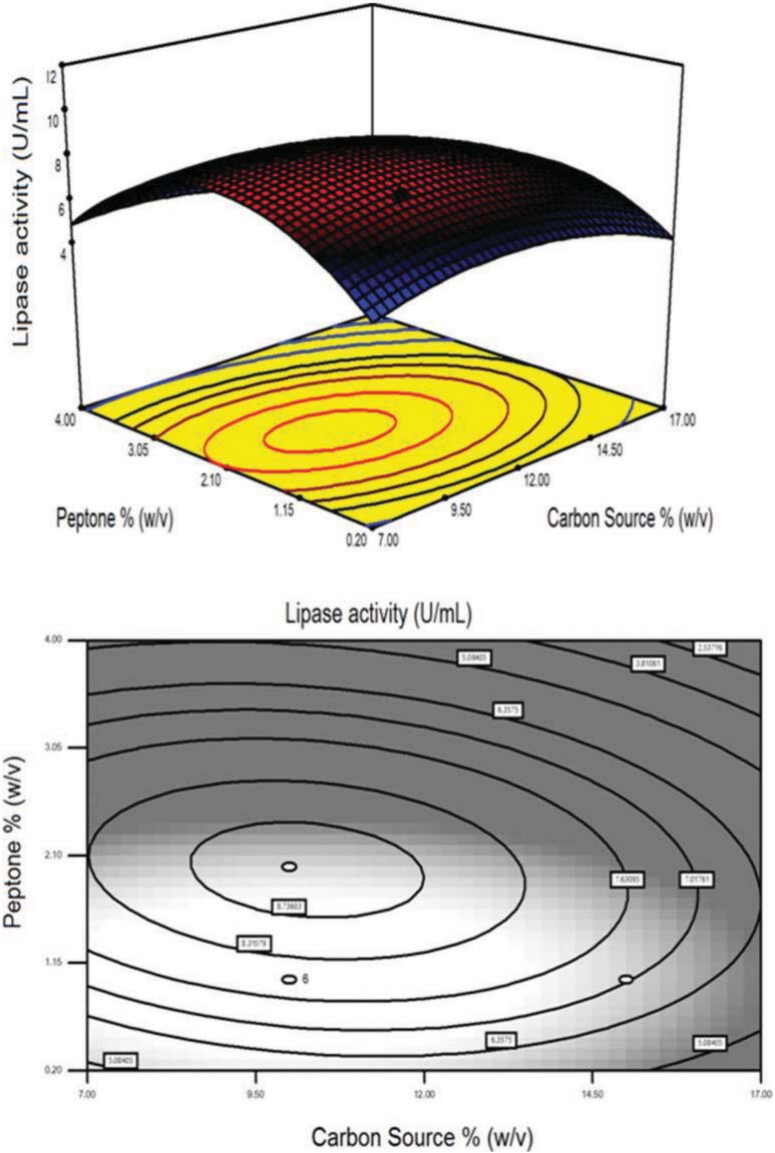
Response surface plot and the corresponding contour plot representing the effects of carbon source and peptone concentration on lipase activity at a fixed ammonium sulphate concentration of 0.5% (w/v).

**Figure 4.**
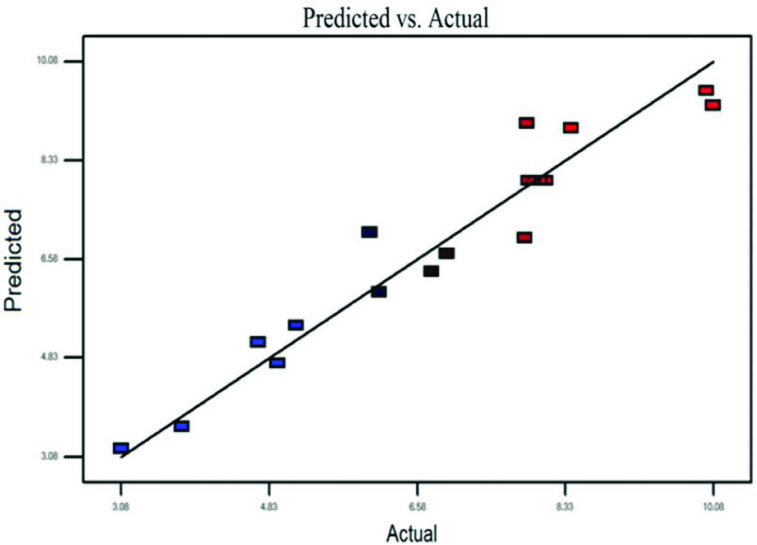
Predicted lipase activity versus experimentally observed lipase activity.

The effect of each parameter on lipase activity and interaction between the three variables were illustrated in Figures 1–4. Depending on the quadratic model, three-dimensional response surface and two-dimensional contour plots were arranged. Figure 1 represents the combined influence of the carbon source and AS concentration on lipase activity at a fixed peptone concentration of 1.0% (w/v). Carbon is essential for the powerful growth of microorganisms. Different carbon sources such as glucose, maltose, lactose and starch have been used to study their influence on the growth of the organisms and also in the production of enzymes and other substances.[38] As shown in Figure 1, the lipase activity was strongly affected by increasing of the concentration of carbon source and AS at a fixed peptone concentration of 1.0% (w/v). Any increment in concentration towards to AS concentration 0.9% (w/v) and carbon concentration to 12.0% (w/v) significantly increased lipase activity up to 10.8 U mL^−1^ but higher values have negative effects on the response. In some studies carried out by Babu et al. [36] and Liu and Zhang [37], the lipase activity was found to be 2.5 and 27.34 U mL^−1^, respectively. A lipase activity level of 20.8 U mL^−1^ was obtained with olive oil by *Nomuraea rileyi*.[38]

The significant effect of the carbon source was reported to be in the range of 5–30 g L^−1^ for different kinds of cultures. [35,39–41] The evaluated values for both peptone and carbon sources are in agreement with the reported data.[39,41] In this work, peptone and AS source were supplied to the medium to respectively provide the organic and inorganic sources of nitrogen required by the microorganism. Because these chemical materials’ level was seen to have a significant effect on lipase activity, its concentration varied.[40,42,43]

Inorganic and organic nitrogen sources play an important role in regulating the synthesis of hydrolyses. Inorganic nitrogen sources are consumed rapidly and may cause repression of enzyme synthesis because of the formation of ammonium-repressible entities, while organic nitrogen sources can provide amino acids, and many cell growth factors, which are essential for cell metabolism and protein synthesis.[45]

Figure 2 represents the effects of AS and peptone on lipase activity at a fixed carbon source concentration of 10% (w/v). AS was used as inorganic nitrogen source and peptone was used as complex nitrogen source, for supplementation of additional nitrogen.[39,42–45]

The effect of supplementation using nitrogen sources on the production of lipase is illustrated in Figure 2. In this frame, a curvature occurred in the response and the maximum level of lipase activity was achieved. Increments in the AS concentration by 0.9% (w/v) increased the lipase activity, while the same response was obtained in peptone concentration as explained in Figure 1. This figure shows that peptone acted as an essential nitrogen source and its supplementation led to further increases in lipase production.

The effect of peptone concentration and carbon sources are illustrated by a fixed AS concentration of 0.5% (w/v) in Figure 3. The maximum lipase activity was obtained when a curvature occurred in the response. Peptone is an important medium supply and is used as a nitrogen source for micro-organisms.[39] In many studies, peptone was used as the nitrogen source for growing micro-organisms on fermentation media, but the effect of its concentration is rarely explored.[44,45]

The values for lipase activity evaluated using Equation (4) versus the observed values are shown in Figure 4. The figure shows that the predicted lipase activities were consistent with the observed levels of activity. The correlation coefficient *R* ^2^ = 0.93 shows that the suitability exists between the predicted and observed values of lipase activities.

Several repetitions were carried out to check model robustness. It was observed that the differences between control experiments undertaken to check the model did not exceed 5% in all runs. It was revealed that the model was highly representative of the study.

## Conclusions

In the present study, it was indicated that SuOC can be successfully utilized in lipase production by *P. fluorescens* (NRLL B-2641). Besides, the supplementation of these solid substrates with AS and peptone (P) was found to increase the lipase production. The maximum lipase activity achieved was 10.8 U mL^−1^. RSM was successfully applied to optimize medium factors and to study the interaction among medium factors and their contribution. This work is crucially important for those researchers studying the production of lipase. It is expected that the results obtained will be utilized for future studies. The preparation of lipase from agricultural wastes in future would help, to some extent, to prevent environmental pollution resulting from release of waste and residues.

## Nomenclature

ANOVA: Analysis of variance
β_0_: Constant coefficient of
β*_i_*: Linear coefficient of Equation (1)
β*_ii_*: Quadratic coefficient of Equation (1)
β*_ij_*: Interaction coefficient of Equation (1)
CCD: Central composite design
R^2^: Correlation coefficient
*RSM*: Response surface methodology
X_i_: Independent variable
X*_1_*: Carbon source (C)
X*_2_*: Peptone (P)
X*_3_*: Ammonium sulphate (AS)
*SuOC*: Sunflower oil cake
*SSF*: Solid substrate fermentation
*SmF*: Submerged fermentation
